# Identifying allosteric fluctuation transitions between different protein conformational states as applied to Cyclin Dependent Kinase 2

**DOI:** 10.1186/1471-2105-8-45

**Published:** 2007-02-07

**Authors:** Jenny Gu, Philip E Bourne

**Affiliations:** 1 Department of Pharmacology and Biomedical Sciences Graduate Program, University of California San Diego, La Jolla, CA 92093, USA; 2 San Diego Supercomputer Center, University of California San Diego, La Jolla, CA 92093, USA

## Abstract

**Background:**

The mechanisms underlying protein function and associated conformational change are dominated by a series of local entropy fluctuations affecting the global structure yet are mediated by only a few key residues. Transitional Dynamic Analysis (TDA) is a new method to detect these changes in local protein flexibility between different conformations arising from, for example, ligand binding. Additionally, Positional Impact Vertex for Entropy Transfer (PIVET) uses TDA to identify important residue contact changes that have a large impact on global fluctuation. We demonstrate the utility of these methods for Cyclin-dependent kinase 2 (CDK2), a system with crystal structures of this protein in multiple functionally relevant conformations and experimental data revealing the importance of local fluctuation changes for protein function.

**Results:**

TDA and PIVET successfully identified select residues that are responsible for conformation specific regional fluctuation in the activation cycle of Cyclin Dependent Kinase 2 (CDK2). The detected local changes in protein flexibility have been experimentally confirmed to be essential for the regulation and function of the kinase. The methodologies also highlighted possible errors in previous molecular dynamic simulations that need to be resolved in order to understand this key player in cell cycle regulation. Finally, the use of entropy compensation as a possible allosteric mechanism for protein function is reported for CDK2.

**Conclusion:**

The methodologies embodied in TDA and PIVET provide a quick approach to identify local fluctuation change important for protein function and residue contacts that contributes to these changes. Further, these approaches can be used to check for possible errors in protein dynamic simulations and have the potential to facilitate a better understanding of the contribution of entropy to protein allostery and function.

## Background

The traditional view of allostery has been redefined as a consequence of an observed shift in protein conformational preference [1-3] upon allosteric interaction largely influenced by a select set of key residues. This is evidenced through an examination of dihydrofolate reductase using COREX[4], an ensemble-based computational model that generates all probable conformational states adopted by the protein thus revealing local stabilizing and destabilizing regions that facilitate conformational shifts. In another example, the conformational state preference of guanine nucleotide binding proteins impacts the preference for their corresponding binding partners[5,6]. In both cases, it has been found that a select set of key residues has a large impact on conformational preference.

With this expanded view of allostery, the model allows for the consideration of other contributing factors and possible mechanisms such as entropy that was initially proposed by the Cooper-Dryden model[7]. This model states that, in an extreme case, the allosteric nature of proteins can be achieved though a shift in vibrational modes without a conformational change in structure. Associated with this model is the idea of entropy compensation where a decrease in local fluctuation in one region of a protein is compensated by an increase in fluctuation in another distant region. This mechanism was first proposed for adenylate kinase[8] and has since been observed in studies performed on, for example, lysozyme[9], staphylococcal nucleases[10], and Tet repressor[11]. The same phenomenon is also observed during ligand binding to dihydrofolate reductase as modeled by COREX[4].

In earlier work, we showed that flexible regions of functional importance can be detected in proteins using only sequence information[12]. This suggests that there are specific sequence patterns that are evolutionarily selected to facilitate allosteric changes. We extend this work to understand the role of these flexible regions associated with particular conformational changes. This is achieved by developing a new structure-based method named Transitional Dynamics Analysis (TDA) to quickly identify these local large-amplitude fluctuation changes between different structural conformers that are important for protein allostery. The procedure involves normalizing large amplitude fluctuations before making a comparison between different protein conformational states to improve the detection of local regions experiencing a change in fluctuation during processes such as regulation and catalysis.

Similar to COREX, the objective of this work is to identify local stabilizing and destabilizing regions that are necessary for protein function. We investigate the contribution of entropy defined by changes in localized fluctuation. While the methods presented here is not as energetically descriptive compared to the assessment of free energy change provided by COREX, it is a computationally less demanding approach to qualitatively identify local regions with changes in flexibility between different conformational states based on normal modes of protein motion.

In addition to detecting local fluctuation changes, we also created an approach to understand the position-specific contributions to global fluctuation, thereby identifying a select set of key residues having a large impact. These contributions to global fluctuation are particularly important in the study of allostery, where networks of interacting residues have been shown to be important[5,6,13,14]. Thus far these networks of interactions have been identified using a sequence-based approach that requires a large number of homologous sequences to detect co-evolving residues. Here we have created a structure-based approach, PIVET (Positional Impact Vertex for Entropy Transfer), to gauge the long-ranged impact of residue pairs in close structural proximity on protein dynamics.

TDA and PIVET were applied to the Cyclin-Dependent Kinase 2 (CDK2) activation cycle where there are representative crystallographic structures and dynamic data using various experimental techniques for each activation stage [15-21]. As will be shown, the available experimental data supports the findings using TDA and PIVET. The CDK2 activation cycle is regulated by cyclin A and involves a series of binding and phosphorylation events to fully activate the kinase leading to the control of cell proliferation[22,23]. The cycle begins with the two-domain CDK2 enzyme in a closed conformation with subsequent ATP binding, followed by complex formation with cyclin A, dephosphorylation of the glycine rich loop (G-loop), and phosphorylation of the activation loop (T-loop). We will demonstrate the importance of understanding fluctuation changes throughout this cycle and consider the broader implications for protein design. Specifically, identified fluctuation changes will be shown to occur in regions that serve as important sites for catalytic or regulatory roles at each specific activation stage.

The approach presented here offers advantages over current approaches that consider structure-flexibility relationships. First, while structural comparison of alternative conformers is a popular approach that can provide valuable insights into the direction of positional change and detect flexible regions such as hinges, it cannot identify fluctuation changes. Second, comparing experimental temperature factors (B values) from X-ray crystallography may miss important fluctuation changes resulting from limitations in the quality and resolution of the data as well as being a local phenomenon. This limitation will be apparent in the analysis of CDK2. Third, Molecular Dynamics (MD) simulations provide highly detailed fluctuation descriptions that cannot be matched by our approach, but because of computational demands, MD simulations are limited to tens of nanoseconds. The approaches presented here can address fluctuation changes that occur over longer time scales by using a coarse-grained protein dynamic modeling algorithm. Finally, as a consequence of relatively short computational times, the approach can be used in a high-throughput mode addressing the rapid growth in protein structures generated by structural genomics efforts. In summary, TDA and PIVET offer a fast, computationally tractable approach to conduct large-scale analysis of motions to understand the fluctuation changes that correspond to conformational changes.

## Results and discussions

### Transitional Dynamic Analysis conducted on the CDK2 activation cycle

TDA and PIVET are algorithms designed to identify significant changes in protein fluctuations as modeled by the Gaussian Network Model (GNM)[24]. GNM is a coarse-grained approach that makes a good approximation of protein fluctuations using only the Cα atoms as nodes of connectivity. All resolvable atoms are accounted for with the construction of the Kirchoff matrix (see Materials and Methods). Decomposing the inverse of this matrix yields a set of eigenvalues and eigenvectors that describe protein fluctuation partitioned into different modes of motion. This decomposition allows us to concentrate our analysis on the two largest amplitude modes because they have been found to sufficiently describe large global motions in proteins [25-28]. We use these decomposed modes to conduct our analysis.

**Figure 1:**
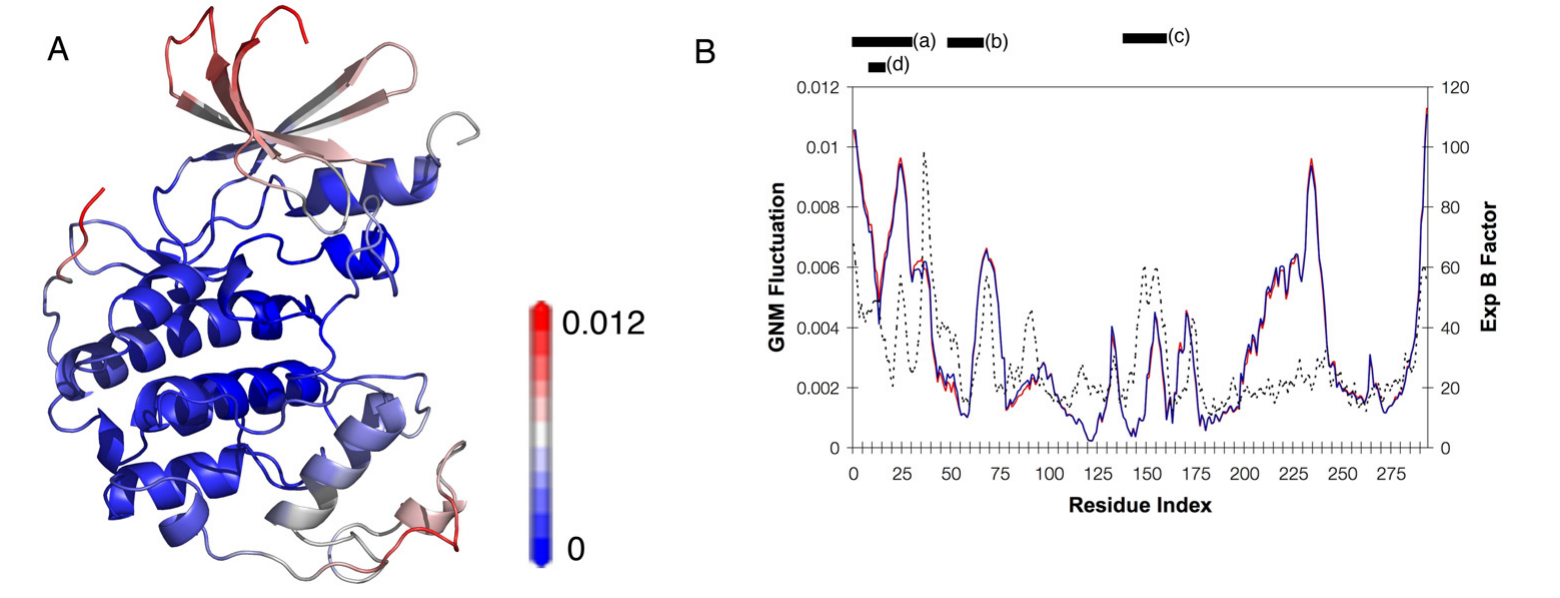
**Comparison of GNM and B Factor Profiles for CDK2**. (A) Structural mapping of GNM dynamic modeling results for the apo form of CDK2 (PDB ID: 1 HCL; see Materials and Methods). The N-terminal lobe (top) is more flexible (red) when compared to the C-terminal lobe (blue). (B) Comparison of modal plots for the two largest amplitude fluctuations are shown for the apo (red) and ATP bound (blue) conformers of ATP. Flexible regions defined by the GNM differs from those defined by experimental temperature factors (dashed line). Locating bars at top represent (a) N-terminal lobe, (b) PSTAIRE helix, (c) T-Loop, and (d) G-loop.

**Figure 2:**
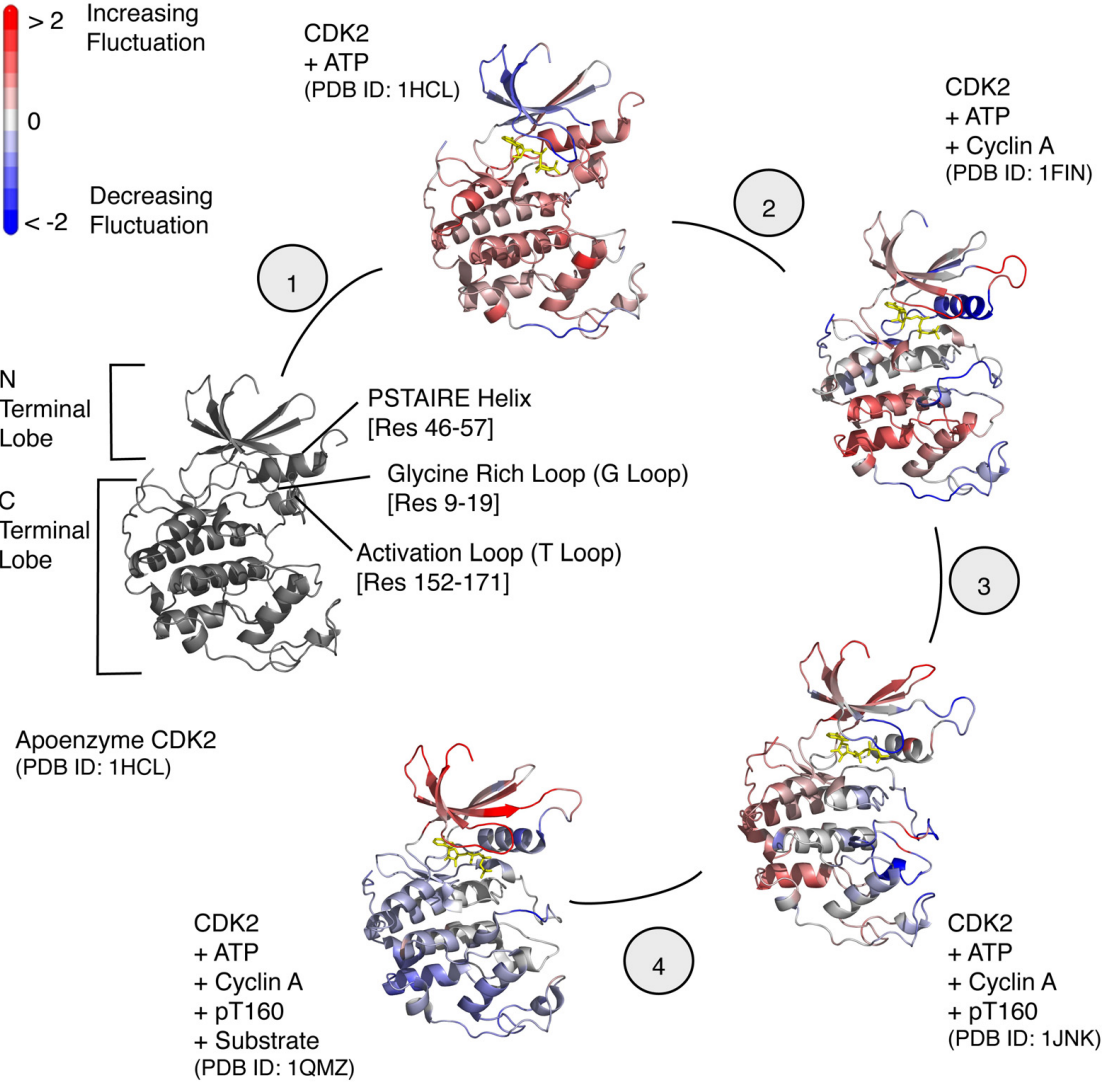
**TDA on the CDK2 Activation Cycle**. Fluctuation changes detected by TDA_*mode *_are mapped to individual structures of the CDK2 activation cycle. To fully activate the kinase, a series of steps must occur involving (1) ATP binding, (2) cyclin A binding (cyclin not shown), and (3) T160 phosphorylation leading to (4) substrate binding (substrate not shown). Values have been normalized such that positive and negative values respectively indicate increasing (red) and decreasing (blue) fluctuations when compared to the previous conformational state. ATP is shown in yellow.

The advantage in analyzing extracted modes over experimental temperature factors (B factors) derived from X-ray crystallography studies is that detected changes only reflect changes in large amplitude fluctuations that are responsible for global motions. Fluctuations arising from higher frequency modes, such as side chain rotations, have little contribution to the global motion and are not considered in this analysis. B factors tend to represent local motions at each atomic position. While there are some agreements in the flexible regions identified by the large-amplitude modes of the GNM and B factor profiles, the descriptions for protein fluctuations are different as observed for CDK2 (Figure 1). With this focus on large-amplitude fluctuation, we conduct TDA on the activation cycle of CDK2 to demonstrate the success of this methodology in identifying fluctuation changes that are important, possibly mechanistically, for protein function (Figure 2). While this method is limited in providing quantitative insights into protein flexibility, it provides qualitative identification of regions with significant changes in fluctuation that are presumed to represent a functional role.

The first step in CDK2 activation is the binding of ATP to the monomer. Structurally the apoenzyme and the ATP bound conformation are very similar with an overall RMSD of 0.39 Å excluding residues 37–40 which are not resolved in either structure[19]. Within the ATP binding pocket, residue conformations are mostly conserved despite the presence of ATP. GNM shows large global fluctuations are mostly localized to the N-terminal lobe with a distinctive stable core in the CDK2 apoenzyme (Figure 1A). The shape of the GNM plot between apoenzyme and ATP bound complex were also found to be highly similar (Figure 1B). This finding is expected since previous modeling efforts with GNM illustrated that proteins with similar architectures employ similar mechanistic modes[29].

**Figure 3:**
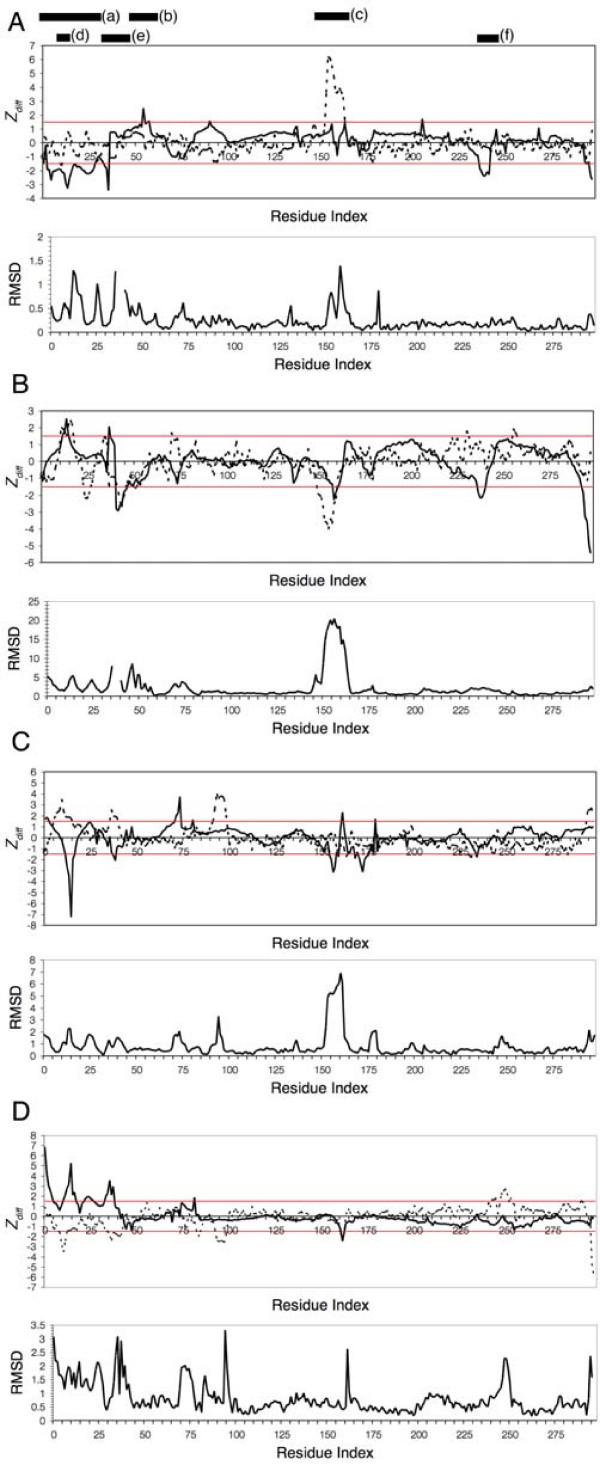
**Fluctuation and Structural Changes Detected in the Activation Cycle of CDK2**. Deviation of fluctuation changes (Z_*diff*_) between different CDK2 conformers: (A) apo and ATP bound structure, (B) ATP bound and cyclin binding, (C) phosphorylated and unphosphorylated T160, and (D) fully activated CDK2 and substrate bound conformer. Structural and fluctuation changes are plotted separately against the residue index of CDK2 only. Different fluctuation changes are observed for the kinase when comparing large amplitude modes (solid lines) and experimental temperature factors (dashed lines) between conformers. Significant changes are identified based on the threshold of 1.5 standard deviations from the mean fluctuation centered at zero (red lines). Structural changes are measured by RMSD between Cα atoms. At top, black bars mark regions of particular interest: (a) N-terminal lobe, (b) PSTAIRE Helix, (c) T-Loop, (d) G-Loop, (e) residues 34–47 important for phosphoryl transfer as well as substrate binding, and finally (f) the CDK insert that is an important binding site for other regulatory proteins.

However, despite similarities in structure and modal shape, TDA_*mode *_(see Materials and Methods) reveals regional changes in dynamics with functional importance for the kinase (Figure 3A). Significant changes in the dynamics identified by TDA_*mode *_between apo and ATP bound form of CDK2 are localized to the N-terminal lobe in the first activation stage. The apoenzyme displays a suppressed PSTAIRE helix (residues 46–57) with an increase in fluctuation located to the N-terminal lobe, particularly the G-loop (residues 9–19), as well as residues 242–246. These changes indicate that ATP binding leads to the destabilization of the PSTAIRE helix and stabilization of the G-loop (Figure 2). The PSTAIRE helix is an important binding site for cyclin A to allosterically regulate this kinase.

In contrast to TDA_*mode*_, performing the same analysis using temperature factors (TDA_*Bfactor*_) (see Materials and Methods) identifies fluctuation changes localized to the T-loop (residues 152–171) while the N-terminal lobe shows no change (Figure 3A). One reason for this disagreement may be that the GNM does not adequately model the temperature factors in the T-loop when compared to the experimental values for the apo and ATP-bound conformers (data not shown). Experimental temperature factors show that the T-loop is more flexible than that was calculated by the GNM therefore indicating that the large amplitude fluctuation is poorly modeled in this region. However, the detection of significant changes in the T-loop at later stages of CDK2 activation is not precluded. Conversely, we find that TDA_*mode *_identifies functionally important fluctuations that were missed by TDA_*Bfactor*_, including effects on the N-terminal lobe upon ATP binding. The implication is that TDA_*mode *_identifies activation stage-specific fluctuation changes important for function. That is, ATP binding does not significantly alter the fluctuation state of the T-loop at this stage. Instead, the fluctuation change in the overall N-terminal lobe is identified to be more important with changes in the T-loop being more significant at later stages.

Following ATP binding, the inactive monomeric CDK2 is then partially activated with the binding of cyclin A that displaces the T-loop by 20Å to open the catalytic cleft to allow for substrate binding[16] (Figure 3B). TDA_*mode *_between CDK2-ATP and CDK2-ATP-cyclin A shows an increase in G-loop fluctuation countered by stabilization in the PSTAIRE helix, T-loop, residues 238–242 and the C terminal tail. Changes in the major functional regions are in agreement with fluctuation changes identified using temperature factors between these two conformers. Despite a poor correlation between calculated and experimental temperature factors in the T-loop as discussed earlier, using mode information, TDA_*mode *_identifies a decrease in large-amplitude fluctuation in this region. Experimental studies show that phosphorylation of the T-loop does not occur until after CDK2 is bound to cyclin A[30,31] therefore suggesting that TDA, with large-amplitude modes, identifies changes in fluctuation when the change is necessary for a particular stage of activation.

To fully activate the kinase after binding of cyclin A, phosphorylation of T160 is required to structurally shift the T-loop for optimal ATP alignment and substrate stabilization leading to subsequent phosphoryl transfer[18]. The resulting stabilizing effect is also detected by circular dichroism and isothermal titration calorimetry[32]. However, other experimental data suggest that T160 phosphorylation, results in a more flexible and disordered T-loop[21,33], irrespective of the presence of cyclin A. This contradictory data can be explained by TDA_*mode *_which shows fluctuation changes in the T-loop, decreasing at the outer edges of the loop while increasing at the center, peaking at residue 162 (Figure 3C) upon phosphorylation. Similar changes were not observed with TDA_*Bfactor*_. Rather, TDA_*Bfactor *_shows changes in a different part of the molecule, with increasing fluctuations for residues 6–15 and 36–40 along with decreasing fluctuations in residues 93–99. These fluctuation changes for the G-loop (residues 9–19) and residues 36–40 disagree with those reported by TDA_*mode *_which shows a decrease in fluctuation of the G-loop and residues 37–38 accompanied by an increase in fluctuation for residues 72–74 and 81. TDA_*mode *_also disagrees with previous MD simulation results and we will return to discuss these disagreements later.

The phosphorylated CDK2-cyclin A complex is now structurally primed for substrate binding during the final stage of activation. Minimal changes in protein conformation were observed and the substrate is found to interact only with the larger C-terminal lobe leading to the exposure of Y15 to solvent and phosphorylation[20]. The T-loop, which is already observed to have a decreased amount of fluctuation in the outer edge of the loop when transitioning from semi-active to active state, undergoes further suppression at the center with substrate binding (Figure 3D). The findings here are in agreement with MD simulations that show the T-loop to have decreased fluctuation upon phosphorylation at T160 [30].

The discussion thus far has been mostly focused on the changes in fluctuation observed for the G-loop and T-loop, however, TDA_*mode *_also identifies fluctuations in other regions of functional importance that have been experimentally validated. First, residues 237–242 were found to be stabilized early in the activation cycle with the binding of ATP followed by cyclin A. This region, also referred as the CDK insert because it is not found in other kinases, has been implicated as a binding interface for other diverse proteins such as CksHS1 [34] and KAP [35] that regulate the kinase. MD simulations also show that this region adopts a highly mobile state indicating that this is a site of conformational change [36]. Second, stabilization of the C-terminal tail was also observed at the beginning of the activation cycle with no change in fluctuation in subsequent stages. Third, residues 34–38 were found to have changes in fluctuation during the later stages of the activation cycle. In a corresponding region of cAMP dependent protein kinase (PKA), the B-helix is observed to undergo an order-disorder transition associated with the phosphoryl transfer process [37]. This transition in PKA was detected by monitoring the backbone flexibility using time-resolved fluorescence anisotropy and suggests that the internal entropy found in this region contributes to the catalytic process. Based on homology between PKA and CDK2, our findings suggest the same is true for CDK2. Finally, another region detected by TDA_*mode *_defined by residues 37 and 41–47 shows a decrease in fluctuation with the binding of cyclin A. MD simulations comparing ligand binding effects between CDK2 and CDK4 show that residues 37–44 are disordered with conformational flexibility affecting ligand binding affinities and potencies[38].

### G-loop fluctuation disagreement with Molecular Dynamics simulations

Decreasing fluctuation changes in the G-loop determined by TDA_*mode *_is in disagreement with both TDA_*Bfactor *_and a previous MD simulation study reporting increased fluctuation [39] during the T160 phosphorylation stage to fully activate the kinase. However, the findings presented here should not be discounted for the following reasons. First, a different MD simulation performed for a longer duration (0.25 μs) showed that the activating phosphate has an overall stabilization effect on global fluctuation, including the G-loop [36]. Second, if we consider the sequential order of regulatory events, the 3 ns MD simulation [39] suggests that a decrease in G-loop fluctuation is observed during the binding of ATP to monomeric CDK2 and is followed by a continual increase in fluctuation until the end of the activation cycle. Based on this MD interpretation of G-loop fluctuation, it is not evident when the loop will form ATP stabilizing interactions that are needed during phosphoryl transfer, an event that occurs several stages after ATP binding [40]. Alternatively, TDA_*mode *_identifies G-loop stabilization at two stages, during the initial binding of ATP and the full activation of CDK2 primed for phosphoryl transfer with T160 phosphorylation. Third, from an experimental perspective, structural data shows that Y15 is buried in the active pT160-CDK2-ATP-cyclin A complex [18] and this finding would be incongruous with the idea that the G-loop has an observed increase in fluctuation. Lastly, the G-loop is important for the exclusion of water molecules and positioning the ATP molecule for phosphoryl transfer to substrate[19]. Again, MD simulation that models the G-loop to have a continual increase in fluctuation after the initial binding of ATP is not supported experimentally. In summary, although the 3 ns MD simulation is in agreement with changes identified by experimental temperature factors [16,18], the lack of agreement with other experimental data and our findings suggest that a longer MD simulation should be undertaken to fully understand the dynamics of the G-loop during the activation cycle.

### TDA identifies potential entropy compensation mechanisms in CDK2

**Figure 4:**
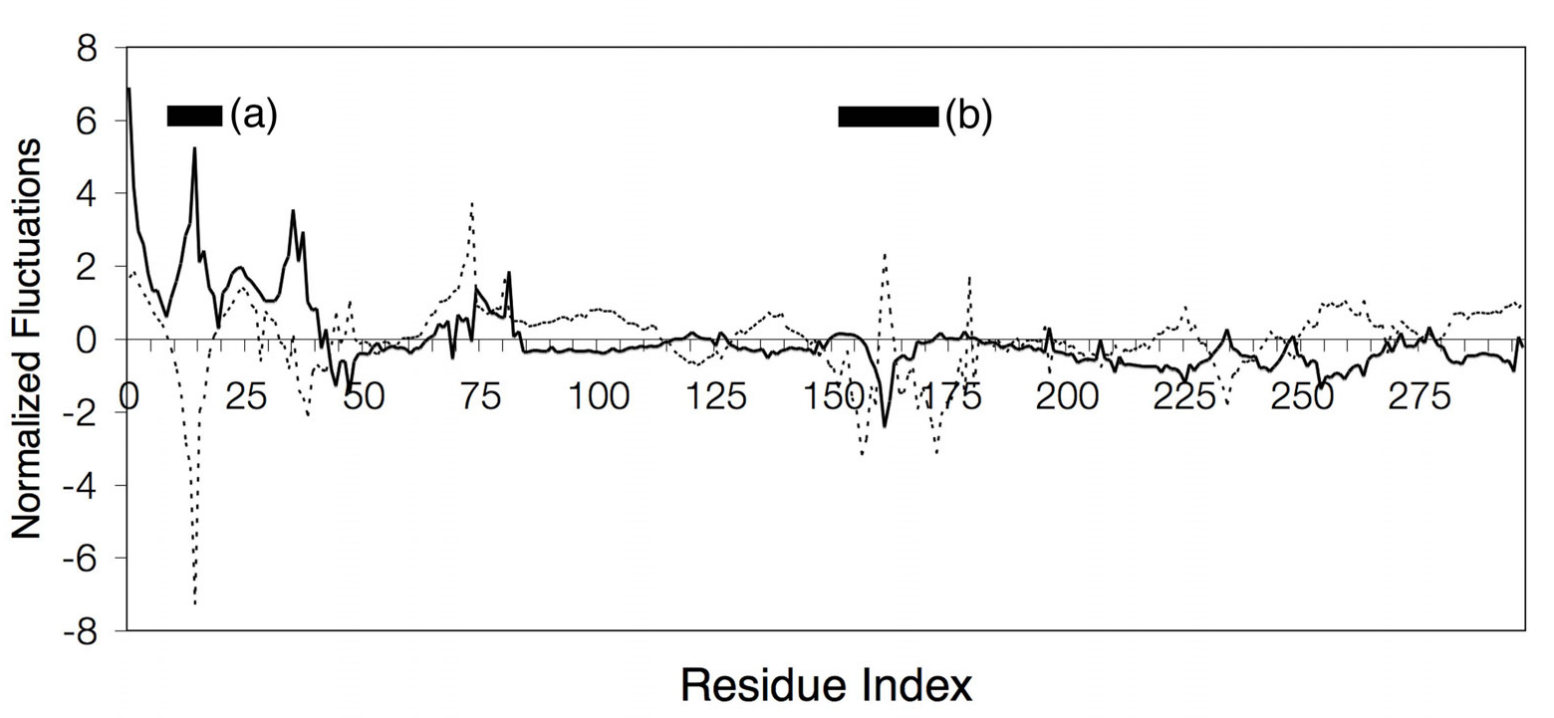
**Entropy Compensation in CDK2**. An example of entropy compensation in CDK2 serving as a possible mechanism for protein allostery. The transitional changes from a semi-active to active state (dashed line) and finally from an active state to substrate bound state (solid line) are shown. The changes in fluctuation between the (a) G-loop and (b) T-loop are observed to have an inverse relationship with each other.

As mentioned earlier, our purpose is to identify fluctuation changes that are functionally important and may have a contributing role to the allosteric nature of the protein. From the results presented here, TDA_*mode *_determined for various stages of the activation cycle of CDK2, suggests that entropy compensation mechanisms are indeed involved. Fluctuation changes for the G-loop and T-loop were observed to be inversely related to each other throughout the activation cycle, most noticeably after the binding of cyclin A. For example, fluctuation changes associated with T160 phosphorylation show a decrease in G-loop fluctuation counterbalanced with an increase in T-loop fluctuation (Figure 4). Upon substrate binding, the G-loop was observed to increase in fluctuation while the T-loop became more stabilized. These changes were not detectable when comparing experimental temperature factors, making TDA_*mode *_a useful approach to quickly identify the contribution of internal entropy, defined by fluctuation changes, to protein function.

### Have protein architectures and functional residues evolved to take advantage of fluctuation changes?

Structural constraints inherently impose a certain amount of evolutionary pressure on sequences [41-43] and we propose that the dynamic restraints needed for function also contribute to this selection and can be identified using TDA. Within the G-loop, TDA_*mode *_identified residues G13, T14 and Y15 to be the three residues with the most dynamic change at different stages of CDK2 activation. Of the three glycines in the Gly-X-Gly-X-X-Gly motif, residue G13 was found to be the most conserved and critical for catalysis [44-46]. Unlike the other glycines of this motif, G13 is also highly conserved in other kinase families besides the typical protein kinase family of which CDK2 is a member [47]. Site directed mutation studies of the corresponding glycine in PKA (G52 in PKA) suggests that this residue serves a structural role by providing the necessary flexibility to interact with ATP [44]. TDA reports G13 to have the largest change in fluctuation of the three glycines in the motif throughout the activation cycle thus highlighting the possible evolutionary pressure imposed by dynamic restraints.

The other two residues, T14 and Y15, are important inhibitory phosphorylation sites in the G-loop that regulate the activity of CDK2 [48]. The dephosphorylation of these residues has been found to be the rate limiting step in activating the kinase [49-51]. From this analysis, TDA has identified T14 to have the most fluctuation change during the first two stages of the activation cycle encompassing the transitions from apoenzyme to ATP bound conformer followed by cyclin binding. Subsequently, Y15 was observed to undergo the most dynamic change during the remaining stages of the activation cycle, full activation of the kinase with T160 phosphorylation and substrate binding.

Lastly, similar observations to those above suggest a possible correlation between the degree of dynamic change and conservation of functionally important residue T160 in the T-loop. This activating phosphorylation site is observed to have one of the most significant fluctuation changes in this region occurring in response to cyclin A binding and phosphorylation to fully activate the kinase. Given that G13, T14, Y15, and T160 are highly conserved it is conceivable that their positioning is part of an architectural design to either maximize or take advantage of the fluctuation change at these sites. Such selective pressure cannot be concluded from a single example, but is worthy of further study.

### PIVET: Identifying important contact changes with long distance effects on fluctuation

To identify the impact of residue pairs on global fluctuation we developed an approach called PIVET (Positional Impact Vertex for Entropy Transfer). First we identify the changing interaction between residue pairs found in two different conformers. Then we conduct serial *in silico *mutations to each of these changing pairs and obtain the resulting large amplitude fluctuation with GNM for comparison to the native dynamic fluctuation (see Materials and Methods). This is achieved through comparison of the Kirchoff matrices (KM) used in the GNM calculation. Since the KM is constructed based on neighboring residues within a given radius threshold surrounding each C-α atom, the residue pairs may not actually be in contact as defined by hydrogen bonding, electrostatic interaction, or van der Waals forces. Therefore this analysis gauges the positional impact for a given protein architecture on global fluctuation based on changes in residue neighbors.

**Figure 5:**
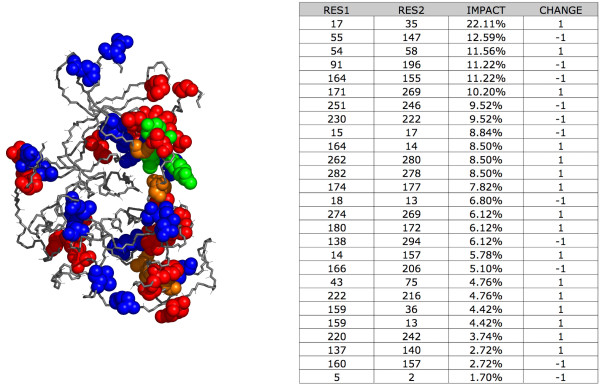
**PIVET Results Between Apoenzyme and ATP Bound CDK2 Conformer**. Residue pair changes between apo and ATP bound conformers of CDK2 and their effect on global fluctuation. 27 residue pairs were observed to have a change in neighboring. Shown are: gain of a neighbor (red), loss of a neighbor (blue), loss of 2 neighbors (green), and no net change observed (orange). The table lists the impact of residue pairs as a percent of overall structure and direction of change where -1 indicate loss of a neighbor (disruption) and 1 a gain of a neighbor in going from the apo to the ATP bound conformer.

For example, comparing residue pair changes between the apo and ATP-bound form of CDK2, we find a total of 27 changes in residue pair neighboring (Figure 5) based on a distance threshold of 7 Å between C-α atoms. The neighboring of residues 17 and 35 was observed to have the most impact on global fluctuation (see Materials and Methods), effecting 22.11% of the protein, while residues 2 and 5 have little to no contribution impacting only 1.70% of total residues.

During the second stage of activation involving the binding of cyclin A to CDK2, 177 changes in pairing relationships were observed in CDK2 with a total of 218 changes including the contacts between CDK2 and cyclin A. Perturbations were conducted only in CDK2 to modify pairing relationship from the ATP bound conformer state to the semi-activated conformer bound to cyclin A. PIVET shows that residues 114 and 142 have the most impact on backbone fluctuations in CDK2 effecting 23.5% of residues with residues 44 and 41 having the least impact (Table 1). The top 10 residue pairs with the most impact on global fluctuation all involve residues within, or in close proximity to, the T-loop and PSTAIRE helix.

**Table 1:** Top 10 Most and Least Impacting Residue Pairs on Global Fluctuation for CDK2

	**TOP 10 PIVET**				**BOTTOM 10 PIVET**			
	
	**Residue Pairs**		**Impact**	**Δ**	**Residue Pairs**		**Impact**	**Δ**
**Activation Stage 2 **(1 HCK to 1 FIN)	A 114	A 142	23.47%	1	A 231	A 238	3.06%	1
	A 156	A 163	22.11%	-1	A 294	A 103	3.06%	1
	A 149	A 146	15.65%	1	A 15	A 36	2.38%	-1
	A 56	A 67	15.31%	-1	A 90	A 296	2.38%	1
	A 151	A 122	14.63%	1	A 163	A 160	2.38%	1
	A 123	A 149	14.63%	1	A 162	A 157	2.04%	-1
	A 122	A 152	14.29%	1	A 295	A 137	1.70%	1
	A 124	A 149	14.29%	1	A 157	A 160	1.36%	-1
	A 151	A 123	13.95%	1	A 36	A 16	1.36%	-1
	A 177	A 158	13.95%	1	A 44	A 41	1.02%	-1

**Activation Stage 3 **(1 FIN to 1 JST)	A 278	A 113	20.81%	1	A 241	A 221	2.35%	1
	A 222	A 216	20.47%	1	B 284	B 287	2.35%	-1
	A 110	A 142	20.47%	1	B 284	B 281	2.01%	-1
	B 310	B 230	19.46%	1	B 372	B 370	2.01%	1
	B 292	A 40	17.11%	-1	B 266	B 271	2.01%	-1
	B 302	B 241	17.11%	1	A 180	A 158	1.68%	-1
	A 158	A 177	16.78%	-1	A 245	A 221	1.68%	1
	A 74	A 38	16.78%	-1	B 389	B 386	1.68%	-1
	B 350	B 390	16.78%	1	B 279	B 284	1.68%	-1
	A 180	A 177	16.44%	1	B 362	B 395	1.01%	-1

**Activation Stage 4 **(1 JST to 1 QMZ)	B 181	A 121	17.11%	-1	A 223	A 228	1.68%	-1
	B 193	B 241	16.11%	-1	B 279	B 284	1.68%	1
	A 37	A 45	15.44%	-1	A 253	A 248	1.01%	1
	A 279	A 283	15.10%	1	B 198	B 202	1.01%	-1
	B 292	A 39	14.77%	-1	B 279	B 291	1.01%	-1
	B 308	B 305	14.77%	-1	B 334	B 359	0.67%	-1
	B 340	B 394	14.77%	-1	B 284	B 287	0.67%	1
	A 113	A 278	14.43%	-1	B 347	B 341	0.67%	1
	B 289	A 40	14.43%	-1	B 419	B 414	0.34%	-1
	A 42	A 38	14.09%	-1	B 391	B 362	0.34%	-1

The 10 residue pairs with the most and least impact on global fluctuation for each activation stage as determined by PIVET. The impact and direction of change for each residue pair with corresponding chains (A and B) are indicated by a loss (-1) and gain (1) of neighbors. Residues are ranked according to their impact on fluctuations in CDK2 only.

Phosphorylation at T160 (1 FIN to 1 JST) resulted in changes between 77 pairs of residues with a total of 143 pairs when including CDK2-cyclin A contacts and those found within cyclin A itself. With substrate binding (1 JST to 1 QMZ), 63 residue pairs were changed in CDK2 with a total of 108 pairs including cyclin A. The interactions between ATP and substrate were not included in this analysis. As expected, positional changes in cyclin A were ranked amongst the lowest impacting residue pairs for these two stages. However, some changes of residue pairs found in cyclin A were ranked amongst the top 10 most influential positions effecting global fluctuation.

In summary, changes in residue pairing have less impact over the course of the activation cycle as the kinase adopts a fully active final conformation. At the start of the cycle with the binding of ATP and Cyclin A, 22.1% and 23.5% of CDK2 residues were impacted. In the final stage, only 17.1% of the residues were affected. This is also expected since these regulation steps, binding of Cyclin A and phosphorylation of T160, serve to stabilize the kinase to catalyze the phosphoryl transfer from ATP to substrate. This analysis is important in providing insight into possible sites sensitive to mutations with a long distance effect on global fluctuation and ultimately protein function. Furthermore, PIVET can also be used to identify potential small molecule binding sites and localize the corresponding impact on protein fluctuation for drug design.

## Conclusion

TDA_*mode *_successfully detects fluctuation changes that correspond to changes in protein conformation located in functionally important regions, while PIVET is able to provide insights into the positional contribution to global fluctuation. The success of both these algorithms has been demonstrated in the activation cycle of CDK2 confirming previous findings while raising the need to revisit another. Both approaches allow us to understand the contribution of fluctuation changes to protein allostery and function by comparing the large amplitude profiles between different conformational states.

Although protein conformations are structurally very similar, TDA_*mode *_was able to identify significant, localized differences in fluctuation profiles as illustrated by comparing the apoenzyme and ATP bound monomeric CDK2. These changes cannot be detected using structure directly or by experimental temperature factor comparisons. TDA requires normalizing fluctuations in a particular mode so that two different protein conformers can be directly compared.

GNM reduces the details of the global protein structure down to just the positional information defined by the Cα atoms, yet it is possible to detect local fluctuation changes in the absence of specific side chain information. As such, without any a priori knowledge, TDA identifies functionally important and highly conserved residues undergoing the largest dynamical changes in that local region. These regions are sensitive to residue changes and have impact on the enzymatic or regulatory function of the protein.

PIVET identifies and gauges the impact of residue pairs on global fluctuation. Similar to the recent discovery of interacting networks facilitating allostery [5,6,13], we show that there are sensitive hotspots in the protein structure that have an impact on global fluctuation. While the role of these residues remains to be confirmed experimentally, the modulation of global fluctuation with this small subset of residue pairs could ultimately modulate protein function.

There are several advantages of using approaches based on coarse-grained protein dynamic modeling algorithms over MD simulations. Large-scale analysis can be conducted with TDA and PIVET to address global fluctuation changes occurring at longer time scales. Compared to MD, the approaches presented here are computationally fast, captures protein motions on a larger time scale, and do not require proteins to be at a global minimum energy state. Conceivably, mode information obtained from any coarse-grained approach can be used to perform TDA but the effectiveness must be tested. GNM is a modeling technique that accounts for all resolvable residues in the protein structure and allows us to focus on backbone fluctuations. Understanding protein dynamics with methods presented here will help guide experiments by identifying target regions for study. With the growing number of available structures, both TDA and PIVET will be especially useful in conducting large-scale analyses between protein conformations.

## Methods

### Gaussian Network Model

The Gaussian Network Model (GNM) is a coarse grain model using only the positions of Cα atoms in a protein structure to model protein fluctuations. GNM has roots in polymer network theory and involves taking the inverse of a Kirchoff matrix Γ where:

Γij{−1if i≠j and Rij≤rc0if i≠j and Rij≥rc−∑i,i≠jΓijif i=j}
 MathType@MTEF@5@5@+=feaafiart1ev1aaatCvAUfKttLearuWrP9MDH5MBPbIqV92AaeXatLxBI9gBaebbnrfifHhDYfgasaacH8akY=wiFfYdH8Gipec8Eeeu0xXdbba9frFj0=OqFfea0dXdd9vqai=hGuQ8kuc9pgc9s8qqaq=dirpe0xb9q8qiLsFr0=vr0=vr0dc8meaabaqaciaacaGaaeqabaqabeGadaaakeaacqqHtoWrdaWgaaWcbaGaemyAaKMaemOAaOgabeaakmaacmqabaqbaeaabmGaaaqaaiabgkHiTiabigdaXaqaaiabbMgaPjabbAgaMjabbccaGiabdMgaPjabgcMi5kabdQgaQjabbccaGiabbggaHjabb6gaUjabbsgaKjabbccaGiabbkfasnaaBaaaleaacqWGPbqAcqWGQbGAaeqaaOGaeyizImQaeeOCai3aaSbaaSqaaiabbogaJbqabaaakeaacqaIWaamaeaacqqGPbqAcqqGMbGzcqqGGaaicqWGPbqAcqGHGjsUcqWGQbGAcqqGGaaicqqGHbqycqqGUbGBcqqGKbazcqqGGaaicqqGsbGudaWgaaWcbaGaemyAaKMaemOAaOgabeaakiabgwMiZkabbkhaYnaaBaaaleaacqqGJbWyaeqaaaGcbaGaeyOeI0YaaabuaeaacqqHtoWrdaWgaaWcbaGaemyAaKMaemOAaOgabeaaaeaacqWGPbqAcqGGSaalcqWGPbqAcqGHGjsUcqWGQbGAaeqaniabggHiLdaakeaacqqGPbqAcqqGMbGzcqqGGaaicqWGPbqAcqGH9aqpcqWGQbGAaaaacaGL7bGaayzFaaaaaa@77D6@

and r_c _= 7 Å is the cutoff radius for each position. The correlated fluctuation between two sites at equilibrium is:

〈ΔRi·ΔRj〉=(3kbT2γ)[Γ−1]ij
 MathType@MTEF@5@5@+=feaafiart1ev1aaatCvAUfKttLearuWrP9MDH5MBPbIqV92AaeXatLxBI9gBaebbnrfifHhDYfgasaacH8akY=wiFfYdH8Gipec8Eeeu0xXdbba9frFj0=OqFfea0dXdd9vqai=hGuQ8kuc9pgc9s8qqaq=dirpe0xb9q8qiLsFr0=vr0=vr0dc8meaabaqaciaacaGaaeqabaqabeGadaaakeaadaaadeqaaiabfs5aeHqabiab=jfasnaaBaaaleaacqWGPbqAaeqaaOGaeS4JPFMaeuiLdqKae8Nuai1aaSbaaSqaaiabdQgaQbqabaaakiaawMYicaGLQmcacqGH9aqpdaqadaqaamaaliaabaGaeG4mamJaem4AaS2aaSbaaSqaaiabdkgaIbqabaGccqWGubavaeaacqaIYaGmiiGacqGFZoWzaaaacaGLOaGaayzkaaWaamWaaeaacqqHtoWrdaahaaWcbeqaaiabgkHiTiabigdaXaaaaOGaay5waiaaw2faamaaBaaaleaacqWGPbqAcqWGQbGAaeqaaaaa@4BD2@

where *k*_*b *_is the Boltzmann constant, *T *the absolute temperature and *γ *a single parameter harmonic potential that accounts for the fluctuation of a residue about a mean axis.

Decomposing the inverse matrix yields a set of eigenvalues and eigenvectors representing the breakdown of fluctuation into modes of motion where the sum describes the overall fluctuation for the given protein. The weighted average of the two largest amplitude modes is used for TDA.

### Transitional Dynamic Analysis

TDA is a two-step normalization procedure that identifies regional fluctuation changes between two protein conformations. The first step normalizes the large amplitude fluctuation between two systems to make a comparison and the second step identifies significant changes in fluctuations. The weighted average of the two largest modes of motion, as calculated by the GNM, is used to identify changes in fluctuation (TDA_*mode*_). We also apply this procedure using isotropic temperature factors (B values) derived from the X-ray experiment for comparison (TDA_*Bfactor*_).

The first normalization step is necessary for comparison of backbone fluctuation between two different systems (conformational states). This is achieved by normalizing large amplitude fluctuations with respect to the intrinsic native fluctuation for each system. A median based approach is used to exclude outliers when calculating the mean fluctuation of the protein and standard deviation needed for normalization. The weighted average of the two largest amplitude fluctuations is used. First the displacement (*mad*) from the median fluctuation (*m*_1_) of the protein for each position (*x*) is calculated. Then, a *M *score for each residue is obtained where:

mad=[|x−m1|]median
 MathType@MTEF@5@5@+=feaafiart1ev1aaatCvAUfKttLearuWrP9MDH5MBPbIqV92AaeXatLxBI9gBaebbnrfifHhDYfgasaacH8akY=wiFfYdH8Gipec8Eeeu0xXdbba9frFj0=OqFfea0dXdd9vqai=hGuQ8kuc9pgc9s8qqaq=dirpe0xb9q8qiLsFr0=vr0=vr0dc8meaabaqaciaacaGaaeqabaqabeGadaaakeaacqWGTbqBcqWGHbqycqWGKbazcqGH9aqpdaWadaqaamaaemaabaGaemiEaGNaeyOeI0IaemyBa02aaSbaaSqaaiabigdaXaqabaaakiaawEa7caGLiWoaaiaawUfacaGLDbaadaWgaaWcbaGaemyBa0MaemyzauMaemizaqMaemyAaKMaemyyaeMaemOBa4gabeaaaaa@43F2@

*M *= |0.6745*(*x *- *m*)/*mad*|

Residues with an *M *score greater than 3.5 were considered outliers and excluded from the calculation of the mean (μ_*mode*_) and standard deviation (σ_*mode*_) of the intrinsic fluctuation found for the specific mode. Fluctuations were normalized (S_*norm*_) for each protein as follows:

Snorm=x−μmodeσmode
 MathType@MTEF@5@5@+=feaafiart1ev1aaatCvAUfKttLearuWrP9MDH5MBPbIqV92AaeXatLxBI9gBaebbnrfifHhDYfgasaacH8akY=wiFfYdH8Gipec8Eeeu0xXdbba9frFj0=OqFfea0dXdd9vqai=hGuQ8kuc9pgc9s8qqaq=dirpe0xb9q8qiLsFr0=vr0=vr0dc8meaabaqaciaacaGaaeqabaqabeGadaaakeaacqWGtbWudaWgaaWcbaGaemOBa4Maem4Ba8MaemOCaiNaemyBa0gabeaakiabg2da9maalaaabaGaemiEaGNaeyOeI0ccciGae8hVd02aaSbaaSqaaGqaciab+1gaTjab+9gaVjab+rgaKjabdwgaLbqabaaakeaacqWFdpWCdaWgaaWcbaGae4xBa0Mae43Ba8Mae4hzaqMaemyzaugabeaaaaaaaa@45CF@

The second normalization step is conducted on the difference between normalized fluctuations (*diff*) obtained from the first step to identify regions with significant changes in fluctuation. This is represented as Z scores to identify significant differences in fluctuation where:

Zdiff=diff−μdiffσdiff
 MathType@MTEF@5@5@+=feaafiart1ev1aaatCvAUfKttLearuWrP9MDH5MBPbIqV92AaeXatLxBI9gBaebbnrfifHhDYfgasaacH8akY=wiFfYdH8Gipec8Eeeu0xXdbba9frFj0=OqFfea0dXdd9vqai=hGuQ8kuc9pgc9s8qqaq=dirpe0xb9q8qiLsFr0=vr0=vr0dc8meaabaqaciaacaGaaeqabaqabeGadaaakeaacqWGAbGwdaWgaaWcbaGaemizaqMaemyAaKMaemOzayMaemOzaygabeaakiabg2da9maalaaabaGaemizaqMaemyAaKMaemOzayMaemOzayMaeyOeI0ccciGae8hVd02aaSbaaSqaaiabdsgaKjabdMgaPjabdAgaMjabdAgaMbqabaaakeaacqWFdpWCdaWgaaWcbaGaemizaqMaemyAaKMaemOzayMaemOzaygabeaaaaaaaa@4957@

This transformation shifts the mean difference to 0 (no change in dynamics between the two states) such that positive values indicate an increase in fluctuation from the reference state and negative values indicate a decrease in fluctuation. Regions with Z scores > 2 or < -2 are considered to be important for the conformational change between states. TDA only reports regions with increasing or decreasing changes in dynamics and does not provide quantitative insights regarding the actual magnitude of fluctuation.

### Positional Impact Vertex for Entropy Transfer

Changes in structural proximity between residues (7 Å radius) are detected by comparing Kirchoff matrices constructed for the GNM calculation. Each identified changes were then modified in the Kirchoff matrix for subsequent calculation with the GNM to understand the effects of changing these relationships. Fluctuations obtained with the GNM with these modified systems were compared to the original results to identify changes in global fluctuation using the TDA algorithm. The impact of residue pairs on global fluctuation was ranked by the impact factor (*I*) defined by the ratio of residues with a significant change in fluctuation (*N*_*TDA*_) to the total number of residues in the protein or isolated region of interest (*N*_*residues*_). Since we focus on fluctuation changes in the CDK2 kinase only, we normalize to the length of this protein instead of the combined total number when including cyclin A. (*N*_*residues *_= 298 residues)

I=NTDANresidues
 MathType@MTEF@5@5@+=feaafiart1ev1aaatCvAUfKttLearuWrP9MDH5MBPbIqV92AaeXatLxBI9gBaebbnrfifHhDYfgasaacH8akY=wiFfYdH8Gipec8Eeeu0xXdbba9frFj0=OqFfea0dXdd9vqai=hGuQ8kuc9pgc9s8qqaq=dirpe0xb9q8qiLsFr0=vr0=vr0dc8meaabaqaciaacaGaaeqabaqabeGadaaakeaacqWGjbqscqGH9aqpdaWcaaqaaiabd6eaonaaBaaaleaacqWGubavcqWGebarcqWGbbqqaeqaaaGcbaGaemOta40aaSbaaSqaaiabdkhaYjabdwgaLjabdohaZjabdMgaPjabdsgaKjabdwha1jabdwgaLjabdohaZbqabaaaaaaa@3FE6@

## Authors' contributions

JG participated in the design and execution of the study, and drafted the manuscript. PEB conceived of the study, participated in the design and helped to draft the final manuscript. All authors read and approve the final manuscript.
